# Diversity of mucoid to non-mucoid switch among carbapenemase-producing *Klebsiella pneumoniae*

**DOI:** 10.1186/s12866-020-02007-y

**Published:** 2020-10-27

**Authors:** Adriana Chiarelli, Nicolas Cabanel, Isabelle Rosinski-Chupin, Pengdbamba Dieudonné Zongo, Thierry Naas, Rémy A. Bonnin, Philippe Glaser

**Affiliations:** 1 EERA Unit “Ecology and Evolution of Antibiotic Resistance”, Institut Pasteur - Assistance Publique/Hôpitaux de Paris - University Paris-Saclay, Paris, France; 2 UMR CNRS 3525, 75015 Paris, France; 3 Sorbonne Université, 75015 Paris, France; 4 EA 7361 Structure, dynamic, function and expression of broad-spectrum beta-lactamases”, Faculty of Medicine University Paris-Sud, University Paris-Saclay, Associated French National Reference Center for Antibiotic Resistance: Carbapenemase-producing Enterobacteriaceae, Le Kremlin-Bicêtre, France

**Keywords:** Capsule, Carbapenem, Insertion sequence, Biofilm, *Klebsiella pneumoniae*

## Abstract

**Background:**

*Klebsiella pneumoniae* is a leading cause of intractable hospital-acquired multidrug-resistant infections and carbapenemase-producing *K. pneumoniae* (CP*Kp*) are particularly feared. Most of the clinical isolates produce capsule as a major virulence factor. Recombination events at the capsule locus are frequent and responsible for capsule diversity within *Klebsiella spp*. Capsule diversity may also occur within clonal bacterial populations generating differences in colony aspect. However, little is known about this phenomenon of phenotypic variation in CP*Kp* and its consequences.

**Results:**

Here, we explored the genetic causes of in vitro switching from capsulated, mucoid to non-mucoid, non-capsulated phenotype in eight clinical CP*Kp* isolates. We compared capsulated, mucoid colony variants with one of their non-capsulated, non-mucoid isogenic variant. The two colony variants were distinguished by their appearance on solid medium. Whole genome comparison was used to infer mutations causing phenotypic differences. The frequency of phenotypic switch was strain-dependent and increased along with colony development on plate. We observed, for 72 non-capsulated variants that the loss of the mucoid phenotype correlates with capsule deficiency and diverse genetic events, including transposition of insertion sequences or point mutations, affecting genes belonging to the capsule operon. Reduced or loss of capsular production was associated with various in vitro phenotypic changes, affecting susceptibility to carbapenem but not to colistin, in vitro biofilm formation and autoaggregation.

**Conclusions:**

The different impact of the phenotypic variation among the eight isolates in terms of capsule content, biofilm production and carbapenem susceptibility suggested heterogeneous selective advantage for capsular loss according to the strain and the mutation. Based on our results, we believe that attention should be paid in the phenotypic characterization of CP*Kp* clinical isolates, particularly of traits related to virulence and carbapenem resistance.

**Supplementary information:**

**Supplementary information** accompanies this paper at 10.1186/s12866-020-02007-y.

## Background

*Klebsiella pneumoniae* is a Gram negative, capsulated, non-motile, rod-shaped bacterium commonly found in the gut flora of healthy individuals, but it can be also found in the environment, particularly in soil and water [1]. Of great concern are infections caused by *K. pneumoniae*, mostly targeting the respiratory and urinary tracts and causing life-threatening infections including pneumonia, sepsis, meningitis, and pyogenic liver abscesses, particularly in health-care settings [2]. Colistin and carbapenems are last-line drugs for *K. pneumoniae* infections but *K. pneumoniae* lineages showing resistance to those drugs emerged and disseminated worldwide, challenging the already limited treatment options [3, 4]. World Health Organization (WHO) included carbapenemase-producing *K. pneumoniae* (CP*Kp*) in the list of PRIORITY 1 critical pathogens for new drugs [5]. An understanding of the pathobiology of *K. pneumoniae* infections would help in developing new control strategies and possible vaccines [6]. Several virulence factors, including the lipopolysaccharide (LPS), fimbriae, siderophores and the capsular polysaccharide (CPS), are contributing to *K. pneumoniae’s* ability to thrive in the environment and in the host.

**Fig. 1:**
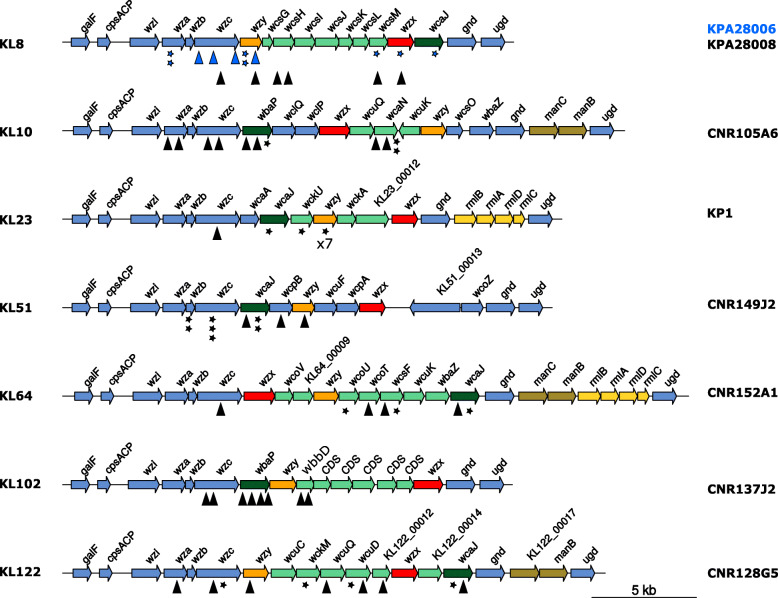
Capsular *loci* maps. Capsular regions from the seven capsular types investigated in this work are represented from *galF* to *ugd*. Genes are indicated by arrows. Blue arrows correspond to core genes from the different capsule loci. Glycosyltransferases are indicated in green. Orange and red arrows represent genes encoding the polymerase and the flippase, respectively. In yellow/dark yellow the genes involved in sugar/repeat unit synthesis (non-conserved). KL numbers are indicated on the left and the analyzed strain of each K-type on the right. IS insertions are represented by triangles and other mutations events by stars as specified in Table 2. Two strains of capsular type KL8 were investigated, blue signs correspond to KPA28006 and black ones to KPA28008

The capsule can ensure resistance to antimicrobial peptides, phagocytosis, and complement-mediated killing [7–10]. More than 77 capsular-types (K-type) have been reported in clinical isolates of *K. pneumoniae* [11]. Large-scale recombination events involving the capsule biosynthesis (cps) region drive an extensive variation, even within the same clonal complex (CC), as described for CC258 isolates [12]. The genes responsible for capsule synthesis are organized into an operon, from *galF* through *cpsACP*, *wzi*, *wza*, *wzb*, *wzc* and *ugd*, with conserved functions and organization and additional glycosyltransferase genes [11] (Fig. 1).

Recent studies shed light on the role of the different genes orchestrating capsule production in *K. pneumoniae*. Mutations in *wza*, *wzb*, *wzc* and *wzy* resulted in reduced amount or absence of capsule, corresponding to a rough, non-mucoid appearance on plate, along with decreased serum resistance and high susceptibility to neutrophilic phagocytosis [13]. On the other hand, inactivation of *wcaJ*, a glycosyltransferase involved in colanic acid synthesis, led to a non-capsulated, non-mucoid variant, which was less efficiently phagocytosed compared to the capsulated *K. pneumoniae*, and which showed a better ability to form biofilm on polystyrene surfaces, and an altered colistin susceptibility [14]. However, capsule production is controlled by a complex regulatory network involving several genes outside the *cps* cluster [15]. Interestingly, loss of mucoid-capsular phenotype has also been reported to occur within the host due to various mutations in the *cps* cluster [16]. Capsule loss due to mutations in the *cps* operon has been shown to occur among clinical ST258 isolates, leading to a better ability to form biofilm and persist in the mice bladder [17]. Altogether, these data suggest an evolutionary advantage that capsule loss can confer to *K. pneumoniae* in the human host and environment under certain circumstances. When analyzed in the laboratory, clinical isolates of *K. pneumoniae* may exhibit mixed populations, noticed by the presence of heterogeneous colony appearance on solid media. This phenomenon may reflect changes in the capsule production within a clonal bacterial population. It is referred as to phenotypic variation, usually from capsulated to non-capsulated variant. However, appearance of colony variants due to changes in capsule biosynthesis might also occur in vitro depending on medium, time and temperature of incubation [18]. This could subsequently lead to the storage and transfer of mixed samples across different clinical laboratories, thus hampering the correct phenotypic characterization of clinical isolates.

Here, we explored the genetic causes of in vitro switching from capsulated, mucoid (M) to non-capsulated, non-mucoid (NM) phenotype in eight clinical CP*Kp* isolates. The frequency of phenotypic switch was strain-dependent and increased along with colony development on plate. The loss of the mucoid phenotype resulted from diverse genetic events: Insertion Sequence (IS) transposition, deletion or point mutations occurring in genes of the *cps* locus. In one variant, a second mutation inactivated *rfaH*, a regulatory gene of capsular polysaccharide biosynthesis [19]. Variation of the impact of the phenotypic switch among the eight isolates in terms of capsule content, biofilm production and carbapenem susceptibility suggested heterogeneous selective advantage for capsular loss according to the strain and the mutations.

## Results

### Non-mucoid variants arise at various frequencies in different *K. pneumoniae* strains

**Fig. 2:**
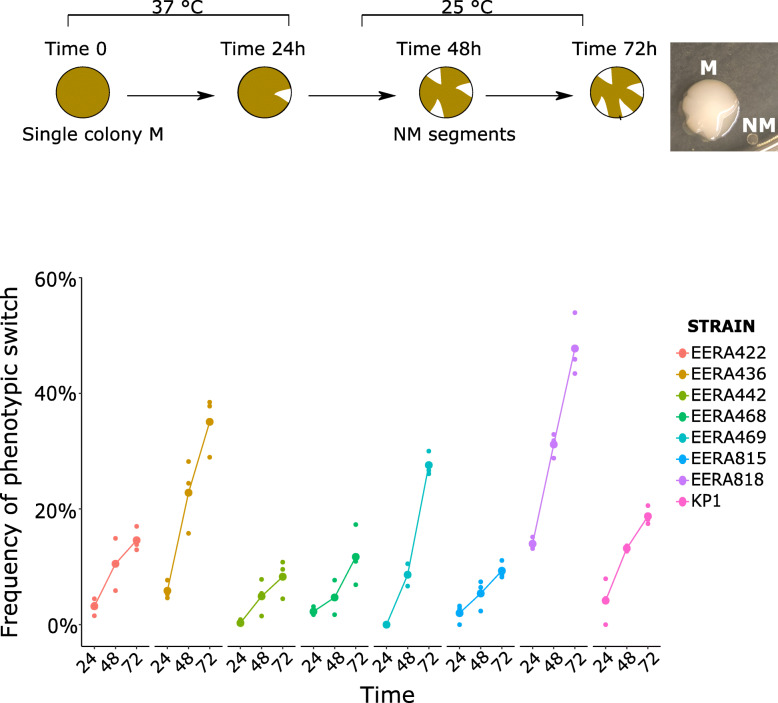
Frequency of phenotypic switch in the eight CP*Kp* isolates. **a**. Left: Workflow followed to visualize and quantify the colony switch from mucoid to non-mucoid phenotype; right: two colonies with NM sectors. **b**. Frequency of phenotypic variation (colony with non-mucoid sectors) monitored over 72 h in the eight CP*Kp* strains. The first 24 h, the isolates were incubated at 37 °C then at 25 °C to observe the appearance of NM segments. M = mucoid, NM = non-mucoid. Strain names are indicated according to the figure key. Frequency and standard deviations are given in Table S4

All along a screening of clinical CP*Kp* strains, we noticed that upon isolation from − 80 °C glycerol stocks, CP*Kp* often exhibited a heterogeneous population with mostly mucoid (M) colonies but also a variable proportion of colonies with a translucent, non-mucoid (NM) appearance on plate. This observation led us to analyze more systematically the population heterogeneity. Hence, we selected eight CP*Kp* isolates belonging to seven STs (ST107, ST16, ST11, ST147, ST383, ST39, ST855) and expressing different carbapenemases (KPC, VIM-1, OXA-48 and OXA-181), as shown in Table 1. The population heterogeneity could be better visualized on TSA medium. We also observed that, when streaked on TSA, M colonies could give rise to either a mixed population (M and NM) or to a pure M population. Furthermore, M colonies might exhibit non-mucoid sectors (Fig. 2a). On the other hand, NM colonies re-isolated on TSA led to homogeneous NM population. The ability of M colonies to produce NM segments was dependent on the incubation time (> 24 h), indicating further selection and growth advantage of NM variants at later growth stages on solid medium.

**Table 1:** Strains analyzed in this work

Strain^**&**^	ST^a^	wzi	K-type^b^	Resistance genes
KP1 [20] SAMN14419408	ST39	wzi83	KL23	**aminoglycoside:** *aac*(6′)-Ib3; *aac*(3)-Iia; aph(6)-Id; **macrolide**: *mph*(E); *msr*(E);** phenicol**: *catA*1; *catB*2;** sulphonamide:** *sul*1;*sul*2; **tetracycline:** *tet*(D); **trimethoprim:** *dfrA*14; *dfrB*1; **β-lactam**: *bla*_SHV-40_; *bla*_OXA-9_; *bla*_SCO-1_; *bla*_VIM-1_; ** fosfomycin:** *fosA*
CNR128G5 SAMN14419403	ST383	wzi162	KL122	**aminoglycoside:** aadA2b; *aac*(3)-IIa; *aac*(6′)-Ib3; *rmtB*;** quinolone:** *oqxA***; sulphonamide:** *sul*1; **tetracycline:** *tet*(A)**; β-lactam:** *bla*_OXA-9_; *bla*_SHV-145_; *bla*_CTX-M-14_; *bla*_CTX-M-15_; *bla*_KPC-2_; *bla*_OXA-1_; *bla*_TEM-1B_**; fosfomycin:** *fosA*
CNR152A1 SAMN14419404	ST147	wzi64	KL64	**aminoglycoside:** *aph*(3″)-Ib; aph(6)-Id**; sulphonamide:** *sul*2; **trimethoprim:** *dfrA*14; **β-lactam:** *bla*_SHV-11_; *bla*_CTX-M-15_; *bla*_KPC-3_; *bla*_TEM-1B_
CNR105A6 SAMN14419407	ST855	wzi100	KL10	**aminoglycoside:** *aac*(6′)-Ib; *aadA*1; *aph*(3″)-Ib; *aph*(6)-Id**; phenicol:** *catA*1; **sulphonamide:** *sul*1; **tetracycline:** *tet*(B)**; β-lactam:** *bla*_SHV-81_; *bla*_KPC-2_; *bla*_OXA-9_; *bla*_TEM-1A_
KPA28006 [21] SAMN14419405	ST11	wzi334	KL8	**aminoglycoside:** *aph*(3′)-VIa; *aadA*1; *aac*(6′)-Iq**; phenicol:** *cmlA*1; **sulphonamide:** *sul*1; *sul*2; **tetracycline:** *tet*(B); *tet*(D)**; trimethoprim:** *dfrA*15; **β-lactam:** *bla*_KPC-2_; *bla*_SHV-182_; *bla*_CTX-M-2_
KPA28008 [21] SAMN14419406	ST11	wzi334	KL8	**aminoglycoside:** *aph*(3′)-Via**; quinolone:** *oqxA*; *oqxB*; **tetracycline:** *tet*(D); **β-lactam:** *bla*_KPC-2_; *bla*_SHV-182_; *bla*_CTX-M-2_
CNR149J2 SAMN14419409	ST16	wzi50	KL51	**aminoglycoside:** *aac*(6′)-Ib-cr; *aadA*2; aph(3′)-Ia**; quinolone:** *qnrS*1; **macrolide:** *mph*(A)**; sulphonamide:** *sul*1; **trimethoprim:** *dfrA*12; **β-lactam:** *bla*_SHV-145_; *bla*_CTX-M-15_; *bla*_OXA-1_; *bla*_OXA-181_; *bla*_TEM-1B_
CNR137J2 SAMN14419410	ST107	wzi173	KL102	**aminoglycoside:** *aac*(3)-Iid; *aph*(3″)-Ib; *aph*(6)-Id**; quinolone:** *qnrS*1**; macrolide:** *mph*(A)**; sulphonamide:** *sul*2**; tetracycline**: *tet*(A); **trimethoprim:** *dfrA*17**; β-lactam:** *bla*_SHV-145_; *bla*_LAP-2_; *bla*_OXA-48_; *bla*_TEM-1B_

&Sequence accession numbers are also indicated, ^a^*ST* = Sequence Type; ^b^K-type = capsular type

In order to determine whether the frequency of switching from M to NM was variable among the selected strains, we quantified colony variants by counting the number of colonies exhibiting non-mucoid segments after 24 h incubation at 37 °C and after 24 and 48 additional hours of incubation at 25 °C (Fig. 2b). Re-isolation of bacteria from these segments confirmed that they led to stable NM colonies. We observed, for the eight strains, a high rate of colony switching, ranging from 10% to more than 40% colonies with NM sectors after 72 h of incubation. We observed the highest frequency (from 15% at 24 h to 47% of the population at 72 h) for *K. pneumoniae* CNR137J2 belonging to ST107 and expressing KL102-type capsule.

### Different mutational events led to a non-mucoid phenotype

To characterize the diversity of the NM variants selected on TSA plates, we isolated 6 to 11 independent NM isolates for each of the eight strains and performed whole genome sequencing (WGS) to compare them with the original isolates. In total, we analyzed 72 NM variants. Libraries were first constructed by tagmentation (>50x coverage). However, we obtained a low or even partial coverage of the AT-rich *cps* locus (Fig. S1), precluding the confident identification of mutations responsible for the NM phenotype. Similarly, Wyres et al. were recently unable to determine the K-type of 129 out of 393 *K. pneumoniae* genomes sequenced by tagmentation [22]. The 72 NM variants were sequenced following enzymatic digestion of the genomic DNA by a mix of nucleases, providing a uniform coverage of the *K. pneumoniae* genome and no discrimination of AT-rich regions (Fig. S1). We identified in all the isolates analyzed, a mutation in the *cps* locus (Fig. 1, Table 2), predicted to be responsible for the NM phenotype. In 26 NM variants, up to five additional mutations were detected elsewhere in the genome. These mutations might have also contributed to the overgrowth of the NM sector (Table S1). In particular, in the NM3 variant of strain CNR149J2 we identified a four-base pair (bp) duplication disrupting the *rfaH* gene, an activator of capsule biosynthesis [19]. Fifty-six percent (40/72) of NM mutants resulted from IS insertion (IS*1*-, IS*L3*-, IS*5*-like) in different essential genes for capsule synthesis (Table 2). In the other variants, we identified point mutations (*n* = 14), including five non-sense mutations or indels (*n* = 18) (Table 2). The genes most frequently affected were *wcaJ* (22.2%), a colanic acid biosynthesis UDP-glucose lipid carrier transferase which mediates the first step of capsule biosynthesis in *Escherichia coli* [23] and plays a role in virulence and phage sensitivity in *K. pneumoniae* [24], followed by *wzc* (20.8%), encoding a putative tyrosine-kinase and *wzy* (15.3%) encoding the polymerase. Other mutated genes encode different glycosyltransferases involved in the complex *cps* synthesis (29.2%) or essential genes like *wza, wzb* and *wzx* (5.6, 2.8 and 2.8%, respectively). In the case of KP1 strain, seven out of the eleven independent NM isolates analyzed shared the same mutation in *wzy*: a deletion of one T in a track of nine T residues, likely resulting from a slippage of the DNA polymerase during chromosome replication.

**Table 2:** Mutations in the capsule synthesis cluster detected in non-mucoid variants

Strain	NM Variant	Gene	Mutation^a, b^
CNR128G5	NM1	*wza*	IS*Kpn25* (pos 382)
	NM2	*wzc*	IS*903b* (pos 2036)
	NM3	*wzc*	G1699T (D567Y)
	**NM4**^c^	*wzy*	IS*Kpn25* (pos 319)
	NM5	*wckM*	Δ4 bp (AAAC) (pos 729); (*)
	NM6	*wcuQ*	IS*Kpn25* (pos 332)
	NM7	*wcuD*	ΔA (pos 38); (*)
	NM8	*wcuD*	IS*Kpn25* (pos 1073)
	NM9	*KL122_00012*	IS*Kpn25* (pos 796)
	NM10	*wcaJ*	insAA^d^ (pos 744); (*)
	NM11	*wcaJ*	IS*1*-like ^e^ (pos 840)
CNR152A1	NM1	*wzc*	IS*Kpn26* (pos 738)
	**NM2**^c^	*wcaJ*	IS*903b* (pos 602)
	NM3	*wcaJ*	T389A (I130N); G452C (G151A)
	NM4	*wcoT*	IS*903b* (pos 866)
	NM5	*wcsF*	IS*903b* (pos 1126)
	NM6	*wcsF*	G118T; (*)
	NM7	*wcoU*	insA^d^ (pos 811); (*)
CNR105A6	NM1	*wza*	IS*1*-like (pos 998)
	NM2	*wza*	IS*1*-like (pos 998)
	NM3	*wzc*	IS*903b* (pos 2073)
	**NM4**^c^	*wzc*	IS*1*-like (pos 542)
	NM5	*wbaP (wcaJ)*	IS*903b* (pos 699)
	NM6	*wbaP (wcaJ)*	IS1-like (pos 316)
	NM7	*wbaP (wcaJ)*	T997G (S333A)
	NM8	*wcaN*	Δ18bp (pos 190–208); (*)
	NM9	*wcaN*	IS*1*-like (pos 358)
	NM10	*wcaN*	IS*1*-like (pos 358)
	NM11	*wcaN*	C79A (V27L)
KPA28006	**NM1**^c^	*wzc*	IS*903b* (pos 715)
	NM2	*wzc*	IS*1*-like (pos 244)
	NM3	*wzc*	IS*1*-like (pos 2146)
	NM4	*wzy*	ΔC (pos 274); (*)
	NM5	*wzy*	ΔC (pos 274); (*)
	NM6	*wcsM*	ΔA (pos 339); (*)
	NM7	*wcaJ*	ΔA (pos 1279); (*)
	NM8	*wzx*	ΔA (pos 422); (*)
	NM9	*wza*	A278T (L93Q)
KPA28008	NM1	*wzc*	IS*1*-like (pos 950)
	NM2	*wzy*	IS*1*-like (pos 319)
	NM3	*wcsM*	IS*903b* (pos 505)
	**NM4**^c^	*wzx*	IS*1*-like (pos 342)
	NM5	*wcsH*	IS*1*-like (pos 605)
	NM6	*wcsH*	IS*1*-like (pos 914)
CNR149J2	NM1	*wzb*	C447A (*)
	NM2	*wzc*	T345A (*)
	**NM3**^c^	*wzc*	Δ11bp (pos 635); (*)
	NM4	*wzc*	G787C (A263P)
	NM5	*wcaJ*	IS*1*-like (pos 905)
	NM6	*wcaJ*	T1145G (L382R)
	NM7	*wcaJ*	A111T (*)
	NM8	*wcpB*	IS*1*-like (pos 347)
	NM9	*wzy*	IS*Kox3* (pos 591)
	NM10	*wzb*	T68G (L23R)
CNR137J2	NM1	*wzc*	IS*903b* (pos 209)
	NM2	*wzc*	IS*1*-like (pos 760)
	NM3	*wbaP (wcaJ)*	IS*1*-like (pos 616)
	NM4	*wbaP (wcaJ)*	IS*1*-like (pos 1312)
	NM5	*wbaP (wcaJ)*	IS*1*-like (pos 247)
	**NM6**^c^	*wbaP (wcaJ)*	IS*1*-like (pos 888)
	NM7	*wbbD*	IS*1*-like (pos 319)
	NM8	*wbbD*	IS*1*-like (pos 284)
KP1	NM1	*wzc*	IS*1*-like (pos 469)
	NM2	*wzy*	T736C (S246P)
	NM3	*wzy*	ΔT (pos 864); (*)
	NM4	*wzy*	ΔT (pos 864); (*)
	NM5	*wzy*	ΔT (pos 857); (*)
	**NM6**^c^	*wzy*	ΔT (pos 864); (*)
	NM7	*wzy*	ΔT (pos 864); (*)
	NM8	*wzy*	ΔT (pos 864); (*)
	NM9	*wcaJ*	A31T; (*)
	NM10	*wckU*	ΔA (pos 16); (*)

^a^In parentheses are indicated: for point mutations, the amino acid change and for other mutations, the position in the gene, ^b^ the star indicates either a non-sense mutation or a frameshift ^c^; NM variant selected for in depth phenotypic analysis (in bold); ^d^ Ins for insertion; ^e^IS1-like correspond to IS*1R*, IS*1SD*, IS*1F,* ISKpn*14* and closely related ISs

### Phenotypic comparison of mucoid and non-mucoid variants

For each CP*Kp* strain, we selected one NM variant with different mutations in *wcaJ, wzc, wzx, wzy* (indicated by the “&” symbol in Table 2) and investigated their impact on phenotypes contributing to their dissemination and persistence in the host.

**Fig. 3:**
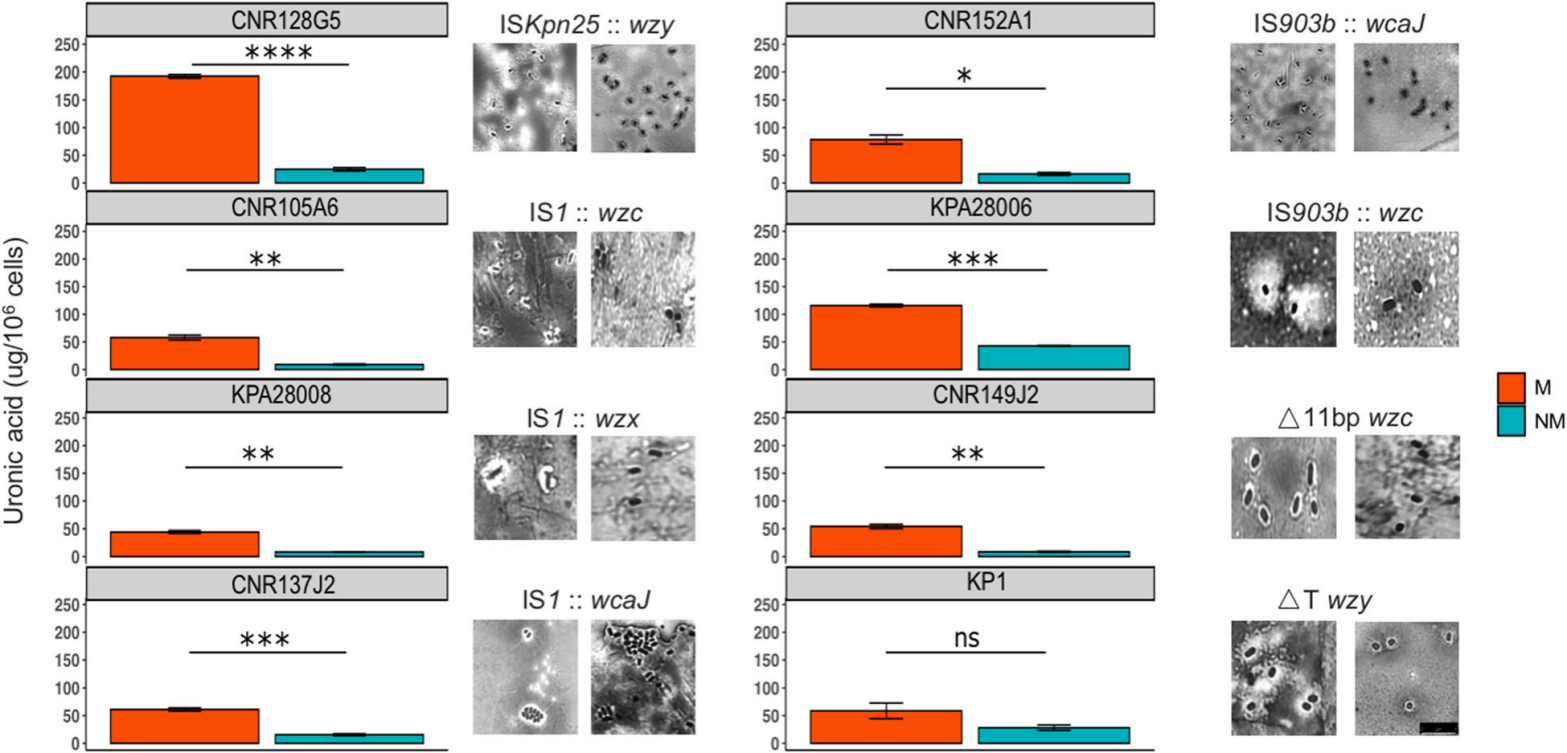
Capsule quantification and visualization in M and NM variants. Uronic acid assay [25] was carried out to quantify capsular polysaccharides in the eight CP*Kp* and their selected NM variants. In all but one couple (KP1), the NM variant exhibited a significant decrease in CPS production compared to the WT isolate. In particular, a stronger difference was noticed in the case of CNR128G5 (*p*-value ****), followed by CNR137J2 and KPA28006. India ink staining was performed to visually detect decrease/absence of capsule in the non-mucoid variant (right image). The scale bar in each image represent 5 μm. Non-mucoid isolates selected for each *K. pneumoniae* strain showed different mutation events (Table 2). M = mucoid, NM = non-mucoid

To evaluate the impact of mutations on capsule production, we quantified uronic acid, a major constituent of the polysaccharidic capsule. In seven out of eight pairs, the NM variant showed a significantly lower amount of uronic acid compared to M variant, confirming that the loss of the mucoid trait resulted from the loss or the reduction of capsule production (Fig. 3). In the remaining strain, KP1, the NM variant (frameshifted in *wzy*), although it did not reach significance, showed a 2-fold decrease in the uronic acid production. The impact on the capsule synthesis in the NM variants was confirmed by capsule visualization by India ink negative staining (Fig. 3).

**Fig. 4:**
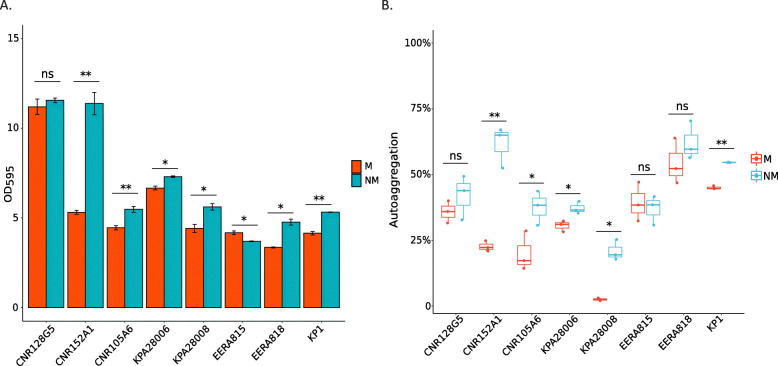
In vitro biofilm production and autoaggregation. **a.** The WT isolates and the selected NM variants were characterized for their ability to produce biofilm in M63 minimal medium supplemented with glucose. In six out of eight couples (CNR152A1, CNR105A6, KPA28006, KPA28008, CNR137J2 and KP1), the NM variant was more efficient in biofilm formation than the parental M strain. Only CNR149J2 showed an opposite trend: the M variant showed a better ability to form biofilm under tested conditions. *p*-values of < 0.001 ***, < 0.01 ** and < 0.05 * were considered significant. **b**. Autoaggregation assay of M and NM variant in spent M63/Glu medium. M = mucoid, NM = non-mucoid are indicated according to the figure key

We investigated and compared the ability of M and NM variants to form biofilm on polystyrene surfaces. For six out of eight strains (CNR152A1, CNR105A6, KPA28006, KPA28008, CNR137J2, and KP1) the NM variants significantly produced more biofilm after 24 h of incubation (Fig. 4a) compared to the parental M strains. However, in the case of CNR149J2 the NM variant produced less biofilm than the parental strain. This opposite phenotypic effect of capsule loss might be related to the mutation in *rfaH*, which has been shown to have a pleiotropic effect [19]. Therefore, the contribution of the capsule to biofilm formation on polystyrene was variable according to strain- and/or mutations.

Autoaggregation might contribute to biofilm formation [26, 27]. The autoaggregative behavior of M and NM derivatives was quantified in spent M63/Glu minimal medium over 24 h at room temperature. Interestingly, all the NM variants but one (CNR149J2) showed, overall, a better ability to autoaggregate under the tested conditions (Fig. 4b). Particularly, the NM variants of CNR152A1, CNR105A6, KPA28006, KPA28008, KP1, which produce more biofilm, showed an increased autoaggregation compared to the parental isolates.

Curli production contributes to biofilm formation and capsule loss can unmask the fimbriae [28]. We compared M and NM isolates for curli production by the Congo red (CR) assay. Among the eight strains analyzed, only one tested positive: CNR128G5, which shows a high level of biofilm formation independently of the M or NM phenotype (Fig. S2). For all the analyzed isolates, we did not observe differences between NM and M variants in the CR assay (Fig. S2), suggesting that capsule loss did not impact significantly on fimbriae production in these isolates.

**Fig. 5:**
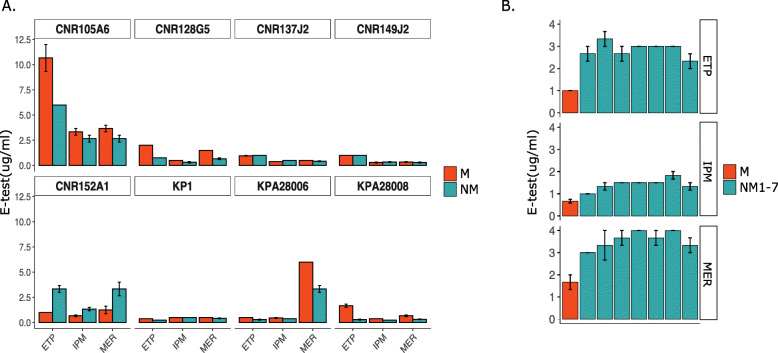
Carbapenem susceptibility profiles of the eight CP*Kp* strains and their NM variants. **a**. The carbapenem susceptibility of the eight CP*Kp* strains and the NM variants was quantified by E-test. ETP = ertapenem; IPM = imipenem and MER = meropenem. Four out of eight NM variants selected showed an increase in susceptibility towards the carbapenem tested, whereas the NM deriving from CNR149J2, CNR137J2 and KP1 did not show any difference compared to the WT mucoid strain. Only CNR152A1-NM2 showed a different behavior, with decreased susceptibility, particularly against meropenem and ertapenem. **b**. Carbapenem susceptibility by E-test of the seven NM variants derivatives of CNR152A1. M = mucoid, NM = non-mucoid. Experiments were performed in triplicates. Error bars represent the standard error of the mean (SEM)

Since the eight strains analyzed harbored a carbapenemase-encoding gene, we compared the susceptibility of both the parental and the NM variants towards carbapenems, which are targeting bacterial cell wall synthesis. Interestingly, we found that minimum inhibitory concentrations (MICs) for the clinically relevant carbapenems (ertapenem, meropenem, imipenem) determined by E-test were different in five out of the eight strains for the M and NM variants. Four NM variants showed an increased susceptibility to those drugs. On the other hand, for CNR152A1, the NM variant (CNR152A1-NM2) - mutated in *wcaJ*, was surprisingly more resistant than the parental M strain (Fig. 5). In order to determine whether this effect resulted exclusively from the mutation in *wcaJ*, genomes were carefully checked for any additional mutation, IS insertion or recombination but we did not identify any in genes known to contribute to β-lactam resistance such as the major porins (*ompK35* and *ompK36*) and their regulator *ompR*/*envZ* and the PBPs (PBP1F, 1A, 1B, 2, 2D). In order to determine whether this effect was isolate or mutation specific, we determined the susceptibility to carbapenems by E-test in the six other independent NM variants from CNR152A1 harboring different mutations in the *cps* locus (Table 2). Consistently with what was observed for NM3, the six other NM variants showed a reduced carbapenem susceptibility. These results confirmed that the reduced susceptibility of CNR152A1 non-mucoid variants was not specific to the *wcaJ* mutant but likely related to the loss of capsule.

Capsule has been shown to play a role in resistance to antimicrobial peptides, including polymyxin B, affecting the interaction with the bacterial outer membrane [29]. Those antibiotics show mode of action and mechanisms of resistance different from ß-lactams. We measured the susceptibility levels of both the parental and the NM variants towards polymyxin B and colistin. However, we did not observe any difference between the M and the NM variant for the eight pairs (Table S2).

## Discussion

Microbial populations inhabit diverse and constantly changing niches, facing oscillations between abundance of nutrients, which allows a prolific bacterial growth, and hostile environments, where individuals adopt strategies to cope with the scarcity of nutrients [30]. One of them is the phenotypic variation, where a population can switch among multiple phenotypes to survive in challenging habitats imposed, for instance, by the antibiotic pressure and the host immune system. In a scenario where multidrug resistant (MDR) clinical isolates are incessantly emerging and spreading, the adoption of such strategies from bacterial populations can further challenge their characterization in the laboratory [31]. *K. pneumoniae* can undergo phenotypic variation, switching from mucoid to non-mucoid colony aspect [32]. Hypo- or non-mucoid appearance has been associated with the loss of capsule in *Klebsiella* and different degrees of mucoidity may indicate diverse mutations affecting capsule synthesis genes [16, 17]. These colony variants might arise in vivo, but also following their isolation in the laboratory. Colony variants can be observed on solid media, but they go frequently overlooked, as they show only subtle differences. Here, by analyzing NM variants deriving from eight clinical CP*Kp*, we showed some phenotypic consequences of the capsule loss potentially contributing to NM variants selection. These phenotypic switches occurred at various frequencies in the different strains analyzed.

By using a newly developed high throughput method called TraDisort, Dorman et al. identified numerous *loci* involved in the regulatory network of capsule synthesis in *K. pneumoniae* [15]*.* However, here we showed that in vitro transition on solid media from M to NM variant (*n* = 72) of eight CP*Kp* clinical isolates is driven by mutations occurring in the capsule locus upon the insertion of IS elements, SNPs and deletions. In a single NM variant (CNR149J2 NM3), we identified both a mutation inactivating *wzc*, which encodes a tyrosine-protein kinase essential for capsule biosynthesis and a mutation in the *rfaH* gene encoding a transcriptional regulator of the *cps* operon [19]. In other NM variants, the disruption of *wzc* led to a non-capsulated phenotype and the contribution of the *rfaH* mutation to the phenotype remains to be determined. Except this specific case, under the conditions we used, mutations in genes involved in this regulatory network, which would reduce capsule synthesis, do not provide a sufficient selective advantage for growth. Recently, Lee et al. observed different colony aspects (mucoid and non-mucoid) among CP*Kp* isolated successively from a single patient [16]. They showed that the within-host evolution of *K. pneumoniae* non-mucoid variants was driven by IS insertion and amino acid alterations in genes essential for capsule synthesis. Likewise, we observed that the NM phenotype among CP*Kp* clinical isolates was related to genetic events happening in the *cps* cluster, particularly targeting *wcaJ*, followed by *wzc* and *wzy* genes. These core genes are essential for capsule synthesis in *E. coli* and *Klebsiella spp.* and their disruption is associated with altered capsule polysaccharide polymerization [14, 15, 17, 33]. However, we identified among the NM variants analyzed, mutations in other genes of the *cps* operon, confirming their contribution to capsule synthesis and the selective advantage for their inactivation. For example, in the strain CNR128G5, the 11 NM variants were mutated in eight different *cps* genes. One exception is the first gene of the operon: *wzi*, as no variant harboring a mutation in this gene was observed. *Wzi* was shown to be an outer-membrane lectin contributing to the formation of the bacterial capsule [34]. Its gene inactivation will probably not lead to a metabolic advantage. However, IS insertion in this gene are predicted to have a polar effect on the downstream genes essential for capsule biosynthesis [35]. The absence of mutation in this gene might indicate a fitness cost of its inactivation under the condition used for capsule mutant selection.

Characterization of selected NM mutants unveiled a diverse landscape of mutations, with the majority (55.6%) linked to IS elements jumping into *cps* genes. Transposable elements are an important source of genetic variability. Movements of ISs are hardly detected using short-read sequencing. Therefore, this type of capsule locus inactivation might be overlooked while analyzing *K. pneumoniae* variants. Transposition is generally maintained at low level to preserve the integrity of the bacterial host genome. However, ISs can undergo bursts of transposition under stress conditions, including oxidative stress and starvation [36], which likely occurred during the prolonged incubation on TSA plates at 25 °C. In the case of KP1, the most frequent event was a thymine deletion in *wzy* (delT), occurring in 6 out of 10 NM independent variants (Table 2). These deletions occurred in a poly(T) track (nt 857–867 of the gene), which could lead to replication slippage, thus increasing the probability of frameshift errors. Similarly, we observed a high rate of frameshift mutations in KPA28006 with three adenosine deletions in *wcsM*, *wcaJ* and *wzx,* corresponding to poly-A track. Interestingly, we also isolated two variants with the same cytosine deletion. This residue is followed by a stretch of a poly-T track. These regions with stretches of nucleotides might be particularly prone to polymerase slippage. The frequency of mucoid to non-mucoid switch in these two strains was among the lowest we have observed. It is possible that the frequency of IS transposition in KP1 (1 out of 11 NM isolates) and KPA28006 (3 out of 9 NM isolates) was lower than in other strains under the condition used, revealing this mechanism of phase variation by increasing its relative frequency. On the other hand, the highest frequency of conversion from M to NM appearance was observed for CNR137J2 (more than 40% over 3-days incubation), followed by CNR152A1 and KPA28008. Notably, in the case of CNR137J2 and KPA28008, all mutants resulted from IS transposition (seven IS*1*-like insertions and one IS*903*b in CNR137J2 and five IS*1*-like insertions and one IS*903b* in KPA28008). One could hypothesize that the high rate of switching in these two strains is due to a higher rate of IS transposition and possibly to IS transposition bursts. In order to assess whether these differences could be related to different occurrence of IS elements in the eight parental strains, we estimated the number of the ISs that were responsible for a non-mucoid phenotype among the 72 isolates we analyzed (Table S3). However, we were not able to correlate the transposition frequency and the number of ISs in the parental strain. Indeed, in the strains KPA28008 and CNR137J2, which contain the smallest number of the analyzed ISs (*n* = 5), all non-mucoid mutants were predicted to result from an IS insertion. On the contrary, in KP1, which contains ten ISs, only one out of the ten non-mucoid variants resulted from an IS transposition.

Here, we also demonstrated that the simple act of picking one colony rather than another one could lead to different interpretations on the phenotype of bacterial isolates from a clinical sample. Indeed, the colony variants exhibited several distinct phenotypic properties in addition to the capsule production, like biofilm formation, autoaggregation and susceptibility to carbapenems. Those phenotypic were predicted to result from altered capsule production. However, additional mutations in some isolates might have also contributed to the observed effect. Particularly, we observed enhanced biofilm production and autoaggregation in M63/Glu for five out of eight NM variants. Higher levels of autoaggregation are indicative of a change in cell surface [26], which here was the loss of capsule, likely unmasking other surface components contributing to autoaggregation. CNR152A1-NM2 showed the highest increase of biofilm formation and autoaggregation, as deduced from the sedimentation rate among the eight strains analyzed (< 24 h), suggesting properties impacting on cell clumping. In-depth analysis of CNR152A1 genome compared to isolates from the same STs retrieved from the National Center for Biotechnology (NCBI) revealed an IS*903b* at codon 264 disrupting *wbbM,* belonging to the antigen O biosynthesis gene cluster. It encodes a glycosyltransferase and its disruption has been shown to induce defects in LPS biosynthesis [37], increasing the electronegative charge of the bacterial cell surface. The IS insertion was also present in the CNR152A1-NM2 and in the other NM variants included in our analysis. The loss of capsule, combined with the mutation in *wbbM*, likely caused a dramatic change from a hydrophilic to a hydrophobic bacterial surface, possibly contributing to enhance the autoaggregative and biofilm formation properties. On the other hand, CNR128G5 produces a high level of biofilm on the whole and no significant difference in biofilm production was observed between NM and M variants.

We also observed a variable impact of the NM phenotype on carbapenem susceptibility. In four out of eight isolates, the capsule loss in the NM variant resulted in an increased susceptibility to meropenem, ertapenem and imipenem. This observation suggested a protection of the mucoid-capsulated phenotype under carbapenem pressure in certain strains, possibly contributing to reduced permeability of the outer membrane to these antibiotics. Interestingly, in CNR152A1, we noticed a decrease in susceptibility in the NM2 variant, particularly towards meropenem and ertapenem. This observation went true for all the NM variants of this strain, regardless the mutated gene. Markedly, loss of O-antigen expression is likely to affect the conformation and function of various surface proteins [38]. Deep rough LPS mutants, lacking the complete core region up to the 3-deoxy-d-manno-octulopyranosic acid residues, were shown to be less capable of assisting the folding, insertion, and trimerization of porins [39]. Hence, we hypothesized that the loss of capsule in the eight CNR152A1 NM variants, combined with the defects in the O-antigen, might further disturb the assembly of outer membrane proteins, resulting in a decrease in carbapenem permeability and susceptibility. Alternatively, autoaggregation and hydrophobicity by themselves might contribute to reduce carbapenem susceptibility. On the other hand, it has been reported that capsular defective strains are more susceptible to antimicrobial peptides including polymyxin B [14, 29]. However, we did not observe the same for the eight strains, possibly due to a strain- and mutation-dependent impact of the capsule loss.

## Conclusions

Capsule loss is a common event, which can take place within monoclonal populations, generating heterogeneity, and it might have an impact on important phenotypic traits related to host adaptation and antimicrobial susceptibility. Given the phenotypic divergences observed between strains, the technical issues encountered to differentiate M and NM variants and to get an optimal sequencing coverage of the capsule biosynthesis region by using tagmentation for library construction, phenotypic characterization of clinical isolates should be performed carefully, especially when those display a multi-drug resistant phenotype.

## Methods

### Strains and growth conditions

The clinical CP*Kp* isolates used in this study were from the National Reference Centre laboratory for Carbapenemase-producing Enterobacteriaceae at the Bicêtre Hospital and are listed in Table 1, together with relevant information: origin, capsular types, ST, antibiotic resistance genes repertoire. *K. pneumoniae* ATCC CIP 53153 was used as a reference strain. Bacteria were grown in Tryptic-Soy (TS) broth or lysogeny broth (LB) and on TS agar (TSA) or LB agar (LBA) plates. Antibiotic susceptibility testing was performed on Mueller-Hinton Agar (MHA) plates. M63 minimal medium was supplemented with Glucose 0.4% (M63/Glu) to perform biofilm assay. Spent M63/Glu medium used to test autoaggregation was recovered following growth of the same isolate, centrifugation at high speed (11,000 x *g*) for 10 min and 0.22 μm filtration of the supernatant.

### Isolation of non-mucoid variants and frequency of phenotypic variation

The M and NM *K. pneumoniae* variants (MVs and NMVs) can be observed on LBA although the NMVs were more accurately distinguished on TSA. We noticed that MVs typically give rise to translucent sectors after 24 h of growth at 37 °C. Appearance of NM sectors within M colonies, referred to as phenotypic switch was quantified as follows. A single mucoid colony was resuspended in normal saline to an OD_600_ of 0.1. One hundred μL of serial dilutions were plated on TSA to get c.a. 100 colonies per plate. After overnight growth, colony aspect (M or NM) was investigated optically by light contrast. The number of mucoid colonies exhibiting non-mucoid sectors was counted after 24, 48 and 72 h of incubation. The frequency of appearance of NMVs was quantified as the ratio between colonies showing NM sectors over the total number of colonies on plate for three independent cultures.

### Capsule extraction and quantification

Capsular polysaccharides were extracted and quantified using a colorimetric assay for uronic acid, a component of the *K. pneumoniae* capsule repeat unit, as described previously [25]. In parallel, serial dilutions of the bacterial culture were plated to determine the number of Colony Forming Units (CFUs). The uronic acid content was expressed in nanograms per 10^6^ CFUs.

### Capsule staining and microscopic visualization

Bacterial capsule was visualized by negative staining with the India Ink method, followed by counter-staining of bacterial cells with Crystal Violet as described [25]. Excess of Crystal Violet was washed away using copper sulfate 10% (wt/vol). Images were taken using a Leica DM-4 B microscope with a PL Fluotar 100x/1.32 PH3 immersion objective and photographed using the Hamamatsu ORCA Flash4.0 LT camera and the Leica Application Suite (LasX) software. The resolution was 2048 × 2048 (72 dpi). No downstream processing was made to enhance image resolution as well as any adjustment and/or manipulation.

### In vitro biofilm production

The ability of the MVs and NMVs to form biofilm on polystyrene was analyzed using 96-well plates [40]. Briefly, stationary-phase cultures were diluted to OD_600_ = 0.05 in fresh M63 minimal medium supplemented with 0.4% glucose and 100 μl of this inoculum were grown for 24 h in 96-well polystyrene plates at 37 °C. Biofilm was fixed by using Bouin’s solution (acetic acid 5%, formaldehyde, 9% and picric acid, 0.9% in water) and washed with water once before adding 1% (wt/vol) crystal violet. After 10 min of staining at room temperature, the plates were washed twice with water, dried at room temperature, crystal violet was solubilized by the addition of 200 μL of a solution of ethanol–acetone (80–20) and OD_595_ was determined. Results correspond to three independent experiments with four-replicates measurements.

### Autoaggregation assay

Autoaggregation (i.e., cell clumping and sedimentation) was measured as previously described [41]. Mucoid isolates and their respective NMVs were grown overnight in M63 supplemented with glucose 0.4% and MgSO4 1 mM at 37 °C shaking. After centrifugation at 3000 *g* for 5 min, bacterial pellets were resuspended in 1 mL of spent M63/Glu minimal medium to get an OD_600_ ~ 0.3–0.6. The OD_600_ of the upper layer of the cultures was read after 24 h upon static incubation at room temperature by an Infinite® 200 PRO spectrophotometer. Autoaggregation percentage was expressed as: (1- OD_600_ upper layer 24 h/OD_600_ bacterial suspension at time zero) *100.

### Curli/cellulase expression

Curli fibers are protease resistant and bind to Congo red (CR) and other amyloid dyes [42]. However, CR binds to other bacterial extracellular features, including cellulose [42]. We therefore assess curli/cellulose production by the Congo red assay. TS agar was supplemented with 40 μg/mL Congo red (Sigma-Aldrich) and 20 μg/mL Coomassie brilliant blue R-250 (Thermo Fisher Scientific). Plates were inoculated with drops of 5 μL of each overnight bacterial culture grown in TSB and incubated for 72 h at 37 °C after which colony morphology and color were inspected and recorded.

### Carbapenem and colistin susceptibility assays

Antimicrobial susceptibility testing was performed by E-test (bioMérieux) on Mueller-Hinton (MH) agar for carbapenems, whereas colistin was tested by broth microdilution assay. Experiments were performed in triplicates. Results were interpreted according to EUCAST guidelines, as updated in 2018 (http://www.eucast.org).

### Whole genome sequencing, genome assembly and variant characterization

Genomic DNA was extracted on exponentially growing *K. pneumoniae* (OD_600_ = 0.4–0.6) using the Qiagen Blood and Tissue DNeasy kit according to manufacturer’s recommendations. Libraries were prepared following manufacturers’ instructions by using the Illumina Flex kit based on Tn*5* transposase tagmentation and the NEBNext Ultra II FS DNA Library prep kit based on random enzymatic fragmentation of genomic DNA. Whole genome sequencing was performed on Illumina NexSeq500 or HiSeq2500 platform. SPAdes [43] was used for de novo assembly and genome annotation was completed using RAST [44]. Resistome, virulome and ST were determined using Kleborate (https://github.com/katholt/Kleborate). SNPs, IS insertion and recombination events were detected by using breseq [45] using as reference contigs of the M variant and in-house scripts based on BWA [46] and GATK [47, 48]. Mutations were visually verified by using the IGV viewer [49]. IS sequences were identified in the draft genome sequences of the eight strains by BLASTN to identify IS – chromosome junctions. IS sequences were retrieved and analyzed by using the ISfinder database [50].

### Statistical analysis

R version 3.6.1 (http://www.rstudio.com/) was used to construct graphs and analyze data regarding phenotypic switch frequencies, capsule quantification, carbapenem susceptibility, biofilm formation and autoaggregation. Groups were compared using unpaired t-test. *P* values of < 0.0001 ****, < 0.001 ***, < 0.01 ** and < 0.05 * were considered significant.

## Supplementary information

**Additional file 1.**

## Data Availability

Fastq files were deposited at the NCBI Sequence Read Archive (SRA) with the BioProject accession number PRJNA613777. Sequence accession numbers of the eight strains are indicated in Table 1.

## Abbreviations

CP*Kp*: Carbapenemase-producing *K. pneumoniae*
LPS: Lipopolysaccharides
CPS: Polysaccharidic capsule
K-type: Capsular-type
CC: Clonal complex
M: Mucoid
NM: Non-mucoid
IS: Insertion sequence
WGS: Whole genome sequencing
SNP: Single nucleotide polymorphism
nt: Nucleotide
TSB: Tryptic-soy broth
LB: Lysogeny broth
CFUs: Colony forming units
wt/vol: Weight/volume
