# Supplementary material for: Diversity of mucoid to non-mucoid switch among carbapenemase-producing Klebsiella pneumoniae
Source: BMC Microbiol. 2020 Oct 27;20:325. doi: 10.1186/s12866-020-02007-y (PMC7590720; doi:10.1186/s12866-020-02007-y)
Supplement: Supplementary file 1 — Additional file 1. [file 12866_2020_2007_MOESM1_ESM.zip › Table S2-4 FigS1-2.pdf]

Supplementary Material

Diversity of mucoid to non-mucoid switch among carbapenemase-producing *Klebsiella pneumoniae*

Adriana Chiarelli[1,2,3], Nicolas Cabanel[1,2], Isabelle Rosinski-Chupin[1,2], Dieudonné Tzongo[1,2], Thierry Naas[1,4], Rémy A. Bonnin,[1,4,#] Philippe Glaser[1,2#*]

Table S1: Additional mutations identified in the nom-mucoid variants (Excel file)

Table S2: Colistin and Polymyxin B MIC of M and of NM variants

Strain	Variant	*Cps* genotype	MIC (µg/ml)[#]	
			Polymyxin B	Colistin
CNR128G5	M	wt	2	4
	NM4	IS*Kpn25 :: wzy*	2	4
CNR152A1	M	wt	2	4
	NM2	IS*903b :: wcaJ*	2	4
CNR105A6	M	wt	2	1
	NM4	IS*1 :: wzc*	2	1
KPA28006	M	wt	2	1
	NM1	IS*903b :: wzc*	2	1
KPA28008	M	wt	2	1
	NM4	IS*1 :: wzx*	2	1
CNR149J2	M	wt	2	1
	NM3	D-11bp *wzc*	2	1
CNR137J2	M	wt	2	2
	NM6	IS*1 :: wcaJ*	2	2
KP1	M	wt	2	2
	NM6	delT *wzy*	2	2

[#] MIC determined by microdilution

Table S3. Estimation of the IS count - for ISs responsible for capsular deficiencies[&].

Strain	ISKpn25	IS*903* – IS*903B*	IS*1*-like[#]	IS*Kpn26*	IS*Kox3*	ISCfr12	IS insertion proportion[§]
CNR128G5	1	5	15	0	3	0	7/11
CNR152A1	0	4	1 (partial)	3	0	0	4/7
CNR105A6	0	4	5	3	0	0	8/11
KPA28006	0	4	5	0	0	2	3/9
KPA28008	0	3	2	0	0	0	6/6
CNR149J2	0	2	4	2	4	0	3/10
CNR137J2	0	2	3	0	0	0	8/8
KP1	0	2	7	1	0	0	1/10

[&] In red IS responsible for capsular deficiency in the corresponding strain; [#] IS1-like corresponds to ISKpn*14*, IS*1R*, IS*1SD*, IS*1F* and closely related ISs; [§]among no-mucoid variants

Table S4: Frequencies and standard deviations of mucoid to non-mucoid switches

Strain	Time point (h)	% NM	Standard Dev.
CNR128G5	24	0.032	0.015
	48	0.105	0.045
	72	0.146	0.021
CNR152A1	24	0.059	0.016
	48	0.228	0.064
	72	0.351	0.053
CNR105A6	24	0.003	0.005
	48	0.049	0.032
	72	0.083	0.034
KPA28006	24	0.023	0.008
	48	0.047	0.030
	72	0.117	0.052
KPA28008	24	0.000	0.000
	48	0.086	0.019
	72	0.276	0.021
CNR149J2	24	0.020	0.017
	48	0.054	0.027
	72	0.093	0.016
CNR137J2	24	0.140	0.010
	48	0.312	0.021
	72	0.478	0.055
KP1	24	0.042	0.040
	48	0.132	0.005
	72	0.187	0.016

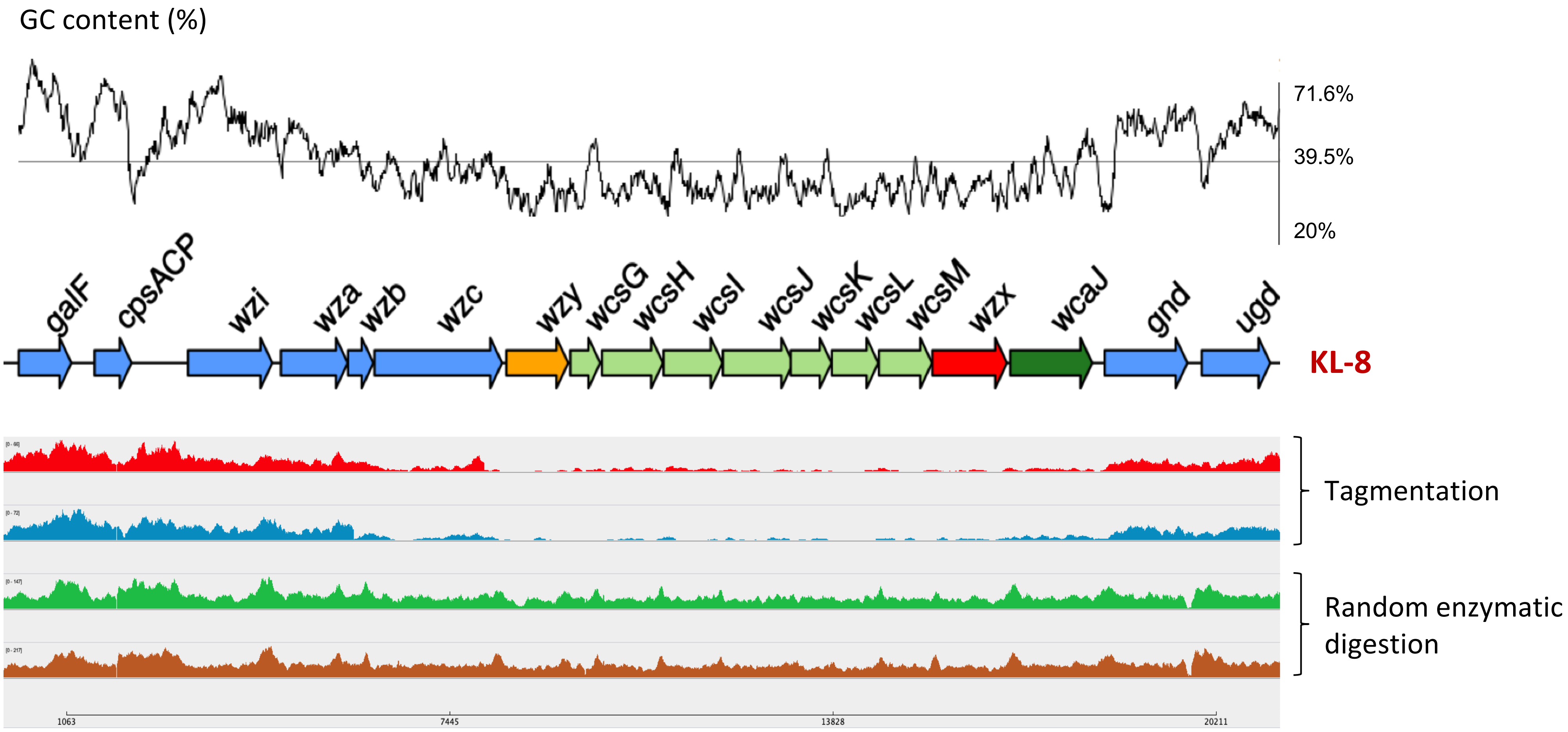

Fig S1. Comparison of sequencing coverage of the capsular region obtained using two different kit for library preparation. The kit based on random enzymatic fragmentation yielded a uniform coverage whereas uneven coverage resulted from sequences prepared with the kit base on transposase tagmentation. Mappings of reads of NM variants from KPA28006 (n=4), belonging to the capsular type KL8, were visualized by using IGV.

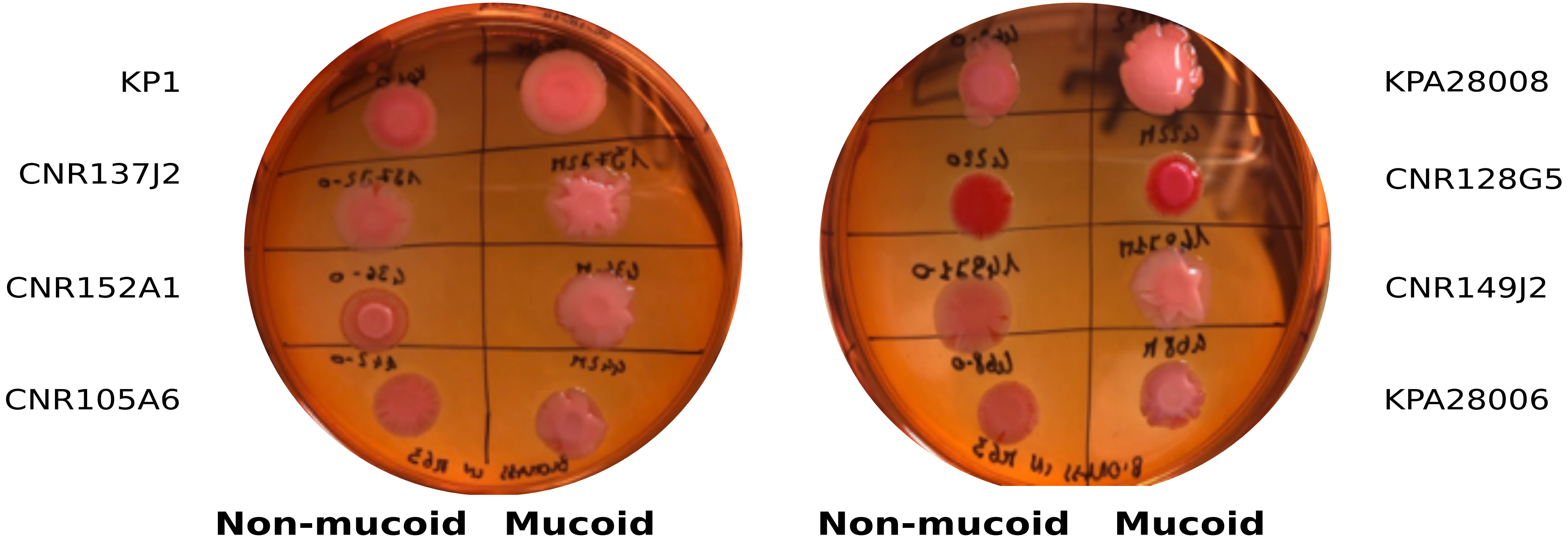

Fig S2. Curli/cellulase expression by Congo red. Colony morphology of the mucoid and non-mucoid variants for the eight isolates of *K. pneumoniae* on Congo red agar.
